# Traumatism Due to Faulty Coordinating Bridgework*Read before the Toronto Dental Society, January 14, 1918, and reprinted from *Good Health* for March.

**Published:** 1918-04

**Authors:** Paul R. Stillman


### TRAUMATISM DUE TO FAULTY COORDINATING BRIDGEWORK*

PAUL R. STILLMAN, D.D.S.

There is an ancient adage which bids the cobbler stick to his last. This axiom is founded on common sense. It might likewise appear, that a specialist in any particular branch should refrain from criticism of other branches

*Read before the Toronto Dental Society, January 14, 1918, and reprinted from *Good Health* for March.

of his subject; that he should adhere to his own line, leaving to others who have practical knowledge and experience any expression of disapprobation. But while the cobbler may not be a tanner he must be able to recognize and procure good leather in order to successfully ply his own trade.

In these days of many specialties it is difficult to draw a positive line of demarcation, so constantly do our subjects overlap and so close is their interdependence. The periodontist, beginning a case, must accept or reject much that is the work of specialists in other branches, and it is the shortcomings of the results in bridgework restoration which most frequently he finds deplorable.

A realization of the growing importance of the subject of the reciprocal relations of the jaws, the occlusion of the teeth as it applies to every dental prophylactic measure and to the articulation of bridgework in particular, is my justification for this paper.

It is quite the universal custom among those who make bridgework to ignore the practical application of accurate measurements of the jaws, which are so necessary to successful results in all restorations for the teeth.

The theoretical principle of substituting for lost natural teeth certain artificial appliances by attaching them to healthy abutment roots is undoubtedly sound. It is the ultimate idea in my opinion, for the restoration of dental function. That cases so treated are followed by disaster, that they are productive of disease and even death, does not prove that the idea is fundamentally incorrect.

In every form of scientific discussion differences arise as to that which is correct and that which is not. The tests of time and usage will in the end show favor to one idea or principle to the exclusion of all others. This is as it should be, for it is by our allegiance to that which has proven good in both theory and practice that scientific advancement is made possible.

The practice of crown and bridgework is at present undergoing a very commendable revolutionary state in its development. New systems and technics have been introduced by various men who have recognized the necessity for greater cleanliness in mouths where bridges were a necessity. Certain forms of removable bridgework appear to have solved the problem of sanitation. Conservation of the normal pulp, and the construction of supplied dummies which represent true anatomical tooth form upon all their surfaces is yet another most commendable advance. Nevertheless the utterly inefficient and worthless type of fixed bridge represented by a row of flat back porcelain facings suspended between two-piece shell or Richmond crowns is still a familiar sight. This type of bridge with its grotesque occlusal surfaces, has always been a menace to hygiene, and the only excuse those who still produce such bridges can offer, is that they were taught the method in their college days twenty years ago.

The periodontist can not with propriety criticize adversely any of the several types of bridges or crowns which are approved by the authorities on this subject, nor the methods and systems of procedure which enter into their construction. It is not the intention of this paper to open discussion on any other phase of bridge or crown restorations than that which deals with the coordinating relation of the jaws in the function of mastication, trauma of the pericementum and its pathological sequelae.

There are certain fundamental essentials in bridgework which apply equally to all operations for the restoration of lost occlusal surfaces of the teeth, whether by bridges, crowns, inlays, fillings or plates, viz., that the occlusal surfaces which are supplied must coordinate with the antagonizing surfaces in the opposite jaw.

Nature has produced for us the plans we are to follow in the restorations which we make for the teeth, and she protests against such work as does not prove up to her requirements, by instituting certain pathological processes in the supporting structures.

That there is a physiological movement of the teeth in the alveoli during normal function is not denied; the investing structures are singularly elastic and attachment by ankylosis in primates would be fatal. That there is a movement of the teeth during the function of mastication, accompanied by malacia is also here affirmed. This normal movement is physiological, always slight, does not increase and is necessary to prevent fracture when marked stress is applied in the occlusion; while the latter is pathological, and is frequently accompanied by evident rarefaction of the investing structures. The physiological movement of the teeth is not easily discernible, as the change of the approximal relation is extremely slight, even when considerable force is applied. The pathological occlusal relation or traumatic occlusion is easily discernible. It is a relation due to a faulty coordination of the occlusal surfaces. It is an acquired condition, and among other causes, is the one I wish to bring to your attention tonight. It may be acquired at the time of the inserting of bridgework and inlays, and it is always as fatal in one case as the other, unless the proper relief is obtained.

The prosthodontist finds that any violation of the laws of dental articulation which has to do with coordination in function is fatal to the practical success of every denture which he makes. Certain difficulties were encountered in the old days, prior to the introduction of anatomical articulation, chief among them the embarrassing knowledge that a denture would adhere to the mucous membrane surfaces upon which it was intended to rest, only so long as the mandible was passive, just as soon as the function of mastication was attempted the plate would become confused with any food bolus, and this resulted in a mastication *of teeth* instead of *by teeth* as was intended. Many of these dentures which in their adaptation to the mucous surfaces were valve tight as to fit, showed a perverse instability when put into service.

The bridgeworker encounters no such unsteadiness after cementing a bridge to place. The bridge is set securely upon two or more abutment teeth or roots, which are presumed to be in normal health. Dynamic stress even at this time is discernible. It is not to be expected that a bridge will gyrate as will a plate which has no abutments to hold it in the mouth—not for a while at least, not until these abutment roots have been literally broken from the alveolar process through exactly the same incorrect mechanical stress relation as in the case of the denture.

The exodontist really believes that a vast majority of teeth are diseased and should be extracted, for it is his experience that most cases which are referred to him actually do need such treatment; while the periodontist seldom sees a case of dental periclasia having bridgework but that it is found necessary to remove (and thus destroy) the bridge in order to proceed with the treatment. Many a handsome and exquisitely made bridge has brought a smile of virtuous pride and satisfaction to both the operator and the patient when it was cemented to place, but there are no laughs and congratulations passed around when the abutments of the thing have been finally destroyed through septic periclasia, and it falls into the hands of the periodontist for removal.

The specialty of bridgework seems to be noticeably unorganized, and the methods of practice quite empirical. Other branches in dentistry which require special talent and training both in the academic and technical aspects, such for instance as orthodontia, radiodontia (Roentgenology) periodontia, etc., have their own organizations which are devoted to the advancement of their several branches; they have journals and published proceedings of their meetings which are national in their scope, and which exert a wide beneficial influence toward a unification of ideas, and a standardization of technic. There are nearly a dozen men of my acquaintance who make bridgework restoration the major branch in practice, yet their individual ideas as to what should be accepted as standard, and what should not would represent almost as many separate ideas as they have faces. There may be many, I hope there are, who construct all bridges and crowns upon the anatomical articulator, and who go to the same scientific measures to produce coordination in the occlusal relations of bridges as do those prosthodontists who construct full dentures upon the principle which Gysi has exemplified to dentistry. I repeat there may be many, but if so, the fact has never been brought to my notice. In the case of bridgeworkers it would appear that the necessity for the proper use of the anatomical articulator seems to be less important to them in an inverse ratio as it appears important to the periodontist.

The scientific procedure of anatomical articulation is a standard method of determining just what the true relation of the teeth should be for each case. One who expects to accomplish satisfactory results in bridgework should discard the antiquated ''rule of thumb'' in articulation, and apply these scientific principles. He should adopt the anatomical articulator with its possibilities for accurate measurements in ascertaining the true relation of the condyle path to the plane of occlusion. He should apply the facebow which will determine just where the casts upon which he expects to build a bridge must be placed in relation to the measurements from the condyles to incisors, and he should never set any bridge where coordination of the occlusal surfaces of the teeth in mastication has not first been fully proven to be accurately normal.

The prosthodontist who does not employ this method in the construction of a denture runs a very high percentage of failure. To be sure the steps necessary for the construction of a denture upon these principles increases somewhat the expense of production, but the constant necessity of remaking these cases which have resulted in failure becomes almost nil.

Dental periclasia has its most frequent and immediate contributing factor in trauma of the pericementum—trauma which is communicated to the investing tissues by an improper relation of the teeth in occlusion. These vascular investing tissues depend upon cell regeneration—a never ending metabolism—for their existence. When trauma is induced, the food supply to these structures is inhibited, malnutrition and unbalanced waste removal follow, vital resistance is lowered, and any break in the integument at the subgingival space is quickly followed by infection.

Normal tissue never becomes infected. There must first occur certain changes which make the tissue pathologically receptive, as well as some break in the integument where the infecting organisms may enter. This is true in regard to the infection of tissue which support the teeth, as elsewhere in the body. In traumatic occlusion the elastic gingival attachment of the dental ligament becomes gradually weakened and finally literally torn from the cervical cementum, opening the subgingival space for the ready entrance of extraneous material. In such an environment the mouth flora find an ideal culture media and a ready passage. Normal gingival epithelum has a very high resistance to all microorganic life, as have all the mucous surfaces of the whole alimentary tract, but when these structures have sustained a prolonged irritation, a constant and determined pounding whenever the jaws come to the closed position, they become exhausted, the natural resistive forces are found too debilitated and disorganized to repel attack. The symptoms of occlusal trauma are to be observed in mouths, perhaps years before the resistance of the investing structures have become so lowered that they become a prey to infection.

The mouth may at times be utterly neglected in so far as its sanitation is concerned, it may even tolerate the presence of badly fitting crown bands with their usual accumulations of septic debris, such a state being endured for years without evidence of periclasia, but let a beautifully carved occlusal inlay or filling be inserted, where zeal for the artistic has resulted in an abnormal cusp relation, or let a piece of bridgework or a single crown be placed in such a mouth and the reciprocal relation of the occlusion violated, forces are immediately set up which result in tissue dystrophy and infection. This would indicate that the immunity of these tissues was sufficient in the one case, where the resistance was high, due to the normality of the occlusal relation, and insufficient in the other, where the abnormal stress relation with its accompanying interference with coordination had reduced the tissue resistance by traumatic occlusion.

Periodontists find bridgework of every conceivable type in the mouths of their patients. A description of some of this should be suppressed—considered as unmentionable in polite dental circles—we will therefore let it pass. A reasonable percentage of this work, however, reveals at a glance that it is the work of the earnest, skillful and painstaking type of practitioner, who comprises the large majority of our profession. Let me present a typical case: overlooking for the moment the well nigh fatally diseased condition of the stumps which are acting as the holding abutments, let us observe the bridge itself. One can well imagine the satisfaction of the dentist when the piece was received from the laboratory quite finished and mounted upon the little plaster of Paris cast representing the segment of the jaws, which was so generously included in the impression. One can see at a glance that the porcelain facings are not checked, nor has the solder which has been so skillfully flowed over the backings and the conventional occlusal swagings, any pits or blow holes. The reinforcement by extra plate and solder makes the shell crowns rather difficult to remove. There is an evident honesty throughout its whole composition. The shade selection of the porcelains is excellent, and they have been ground to fit the gum contour with precision. The buccal resemblance to teeth is striking. This seems as far as we can go in commendation or compliment, for the occlusal surfaces are quite untooth-like in both outline and form. And the lingual surface—there is no lingual surface. From the lingual cusp to the buccal cervical border there is nothing—just a smooth inclined plane to encourage the tongue in its efforts to dislodge food particles. This surface has been called a self-cleansing surface, an example of misnomer of the most pronounced type. Study models of such a case would reveal that all of the occlusal contact stress had been concentrated upon the bridge, for the abutments are loose and elongated, due to a thickening of the pericementum by occlusal trauma. They are also septic. It is today a professional crime to set a bridge such as I have described. Infection of the abutments must inevitably follow in this case, as in that of the tooth with a root canal which has been filled with absorbent cotton. This particular kind of result in bridgework is the outcome of unquestioning allegiance to antiquated methods, together with a disregard and ignorance of the anatomical movements of the mandible.

The making of study models as a forecast of treatment in restoration, is a common practice among many of the more advanced practitioners. These study models are typical orthodontic casts which are occluded in their true relations. Prognosis for the necessary anatomical restorations may thus be studied at leisure, and the scheme definitely decided before treatment is begun. The taking of impressions of finished cases, for the purpose of making casts for criticism of one's own results, is a practice which is indulged in by a very few—yet were this a customary practice what an infinite improvement would soon result. Diagnosis for prophylaxis in such cases as these would reveal traumatic occlusion, if present, and much harm could thus be prevented. Were the results of these failures of bridgework through this fault apparent after a few days or weeks, instead of after several years of unsatisfactory service; were the symptoms which are induced of a painful and inflammatory character from the very first, this paper would never have been written, for, like the prosthodontist, the bridgeworker would have definite and immediate trouble on his hands, and the remedy would have been adopted simultaneously with its introduction into the denture work.

The practice of employing laboratory assistants, or of sending cases to the public laboratories is good, provided one obtains competent workmanship. It must be remembered that these so-called mechanical dentists can only supply technical help, that they have no academic knowledge of the anatomy of the parts which are undergoing replacement, nor do they even have an opportunity to observe in practical service the appliances upon which they work. That they frequently have skill in their work, which exceeds that of the dentists who employ them, is obvious. But so has the bricklayer superior technical skill to that of the architect, and so it should be. That the laboratory man's results are ever a failure is largely the fault of those who employ him. It has been said of the alarm clock that to be successful with its use one must know more than the clock.

In investigating the relation of the dentist to the public laboratory, I have found that it is customary, for the dentist, to send to the laboratory impressions which include never more than the lateral half of the jaw; that the articulator used is of the hinge principle type; or else what is known as the "back extension" is used in place of an articulator, the latter having but one movement, lifting apart as a cover lifts from a box.

It is necessary, in order to obtain results which are satisfactory, to have an entire impression of both jaws, and to have the casts poured in some material which has sufficient hardness on the occlusal edges to withstand attrition while the cast is being articulated. The antagonizing cast should never be made from a wax bite, but from a cast made from an impression and the upper and lower casts then assembled upon the anatomical articulator with the aid of the face-bow.

Bridgework should resemble the natural teeth in so far as it is possible. Correct measurements of the crown diameters should depart from the true measurements but slightly, if at all. Lingual surfaces are of greater importance to the function of mastication than the buccal and labial and they should be reproduced with greater care. Where there is great loss of alveolar tissue in the bicuspid and molar region, lingual roots should be carved upon these surfaces to lighten the weight or thickness, never departing from the true anatomical forms which nature has adopted and which are fundamentally the correct ones.

The anatomy of the tempo-mandibular joints vary in every individual as do the shape of the ears upon the outside of the individual's head. Its development as to form is greatly influenced by the erupting arrangement of the natural teeth in their occlusal relations. Cases of unilateral deformities in malocclusion exhibit marked differences in the curve of the condyle path in this articulation. Certain maxillary habits are formed in each case previous to the loss of the teeth, and in any successful restoration of these lost teeth these facts should be ascertained and taken into consideration. Normal arrangement of the teeth in bridge-restoration will not be tolerated in certain cases of this type when the maxillary habit is greatly interfered with.

There are certain names to which credit for research in this work should be given, for it is by the labor of such men as Bonwill, Snow, Gysi and Williams particularly, and many others, that this science has been developed. One does not claim that there has been suggested here anything original nor admit that the anatomical articulator has not been used in these cases and found successful.

The name of Peeso is almost as familiar to the bridgeworker as is the work itself. Dr. Frederick A. Peeso has recently published a text book, entitled "Crown and Bridgework." It is a complete working compendium for this field. If the plea which I have made here has not impressed you with the gravity of the situation, I can do no better than to quote from the writings of this master of the subject. He states: "Another fundamental to crown and bridgework success was brought to the fore when a few scientifically inclined earnest workers, in an effort to make more effective artificial dentures, began a careful study of the various movements of the mandible during mastication. The object of these efforts was to devise a contrivance to accurately reproduce these movements so as to enable the dental workman when mounting artificial teeth to secure a more normal occlusion. Heretofore, if the upper and the lower teeth articulated with each other when the mandible was at rest, the work was deemed satisfactory, notwithstanding that the dentures were ineffective in mastication, owing to the fact that all the teeth met only when the mandible was in the rest position. At other times but few teeth were in contact. The immediate result of these investigations was a better understanding of the mechanism of mastication and a higher appreciation of the importance of normal occlusion that has since reached all departments of our profession.''

''By occlusion is understood a rubbing or grinding surface contact of all the masticating surfaces of the teeth during all the movements of the mandible as is always the case with the natural teeth in their normal position. Articulation is a mere fitting together in one position only.''

''This understanding of occlusion brought to the fore an imperfectly recognized cause of failure of many dental bridges. With the mouth closed, the teeth on these bridges fitted the opposing teeth accurately, but during mastication they touched at a few points only. Except for this fact these bridges might have given many years of excellent service, but owing to defective occlusion the force of mastication was concentrated upon a few teeth, which resulted in literally pounding the structure to pieces in a short time. In other cases the stress set up a destructive irritation in one or more of the supporting abutments, which just as surely resulted in the bridge failing. We know now that it is impossible for any one tooth to be unduly strained during mastication, or other movements of the mandible if the occlusion of the denture has been properly adjusted, be it a plate, a crown or a bridge.''

This work of Peeso's is of such importance in the literature of bridgework that I will also take the liberty of quoting from this same source from his chapter headed ''Articulation.'' He states: ''In crown and bridgework the question of the occlusion is of most vital importance, as the stability and life of the work depends to a very great extent upon its proper occlusion with the opposing teeth.''

''In all cases of bridgework, it is absolutely essential that only first-class anatomical articulators, capable of reproducing the natural, lateral or trituating movements of the mandible, so necessary for perfect mastication, should be used. * * * Nearly all of the small so-called crown articulators on the market are absolutely worthless so far as securing good results are concerned. With these articulators the only movements possible are simply the up and down, or the opening and closing of the mandible. * * *

''In the majority of cases * * * the face-bow should be used to serve as a guide to mount models properly on the articular.''

The time has arrived when our profession is being looked upon as a group of scientifically trained men who have as their fundamental idea the prevention and cure of all disease which has its inception within the confines of the mouth. To be accredited with less would be abhorrent to every ethical practitioner. To be *producers of disease* instead of physicians, to be *destroyers of teeth* instead of dentists, to be *artisans instead of surgeons* is the very antithesis of our aspiration. So, if there has been a scandalously high rate of failure in our bridgework, let us get together and see that this stain on the escutcheon of the best profession in the world is wiped out. Let all among us who have at heart the high ideals of our profession, either correct the practice of using a hinge articulator or quit making bridgework.

